# Improved Ant Colony Clustering Algorithm and Its Performance Study

**DOI:** 10.1155/2016/4835932

**Published:** 2015-12-29

**Authors:** Wei Gao

**Affiliations:** Key Laboratory of Ministry of Education for Geomechanics and Embankment Engineering, College of Civil and Transportation Engineering, Hohai University, Nanjing 210098, China

## Abstract

Clustering analysis is used in many disciplines and applications; it is an important tool that descriptively identifies homogeneous groups of objects based on attribute values. The ant colony clustering algorithm is a swarm-intelligent method used for clustering problems that is inspired by the behavior of ant colonies that cluster their corpses and sort their larvae. A new abstraction ant colony clustering algorithm using a data combination mechanism is proposed to improve the computational efficiency and accuracy of the ant colony clustering algorithm. The abstraction ant colony clustering algorithm is used to cluster benchmark problems, and its performance is compared with the ant colony clustering algorithm and other methods used in existing literature. Based on similar computational difficulties and complexities, the results show that the abstraction ant colony clustering algorithm produces results that are not only more accurate but also more efficiently determined than the ant colony clustering algorithm and the other methods. Thus, the abstraction ant colony clustering algorithm can be used for efficient multivariate data clustering.

## 1. Introduction

Clustering divides data into homogeneous subgroups, with some details disregarded to simplify the data. Clustering can be viewed as a data modeling technique that provides for concise data summaries. The objective of the division is twofold: data items within one cluster must be similar to each other, whereas those within different clusters should be dissimilar. Problems of this type arise in a variety of disciplines ranging from sociology and psychology to commerce, biology, computer science, and civil engineering. Clustering is thus utilized in many disciplines and plays an important role in a broad range of applications; because of this, clustering algorithms continue to be the subject of active research. Consequently, numerous clustering algorithms exist that can be classified into four major traditional categories: partitioning, hierarchical, density-based, and grid-based clustering methods [1].

The ant-based clustering algorithm is a relatively new method inspired by the clustering of corpses and larval sorting activities observed in actual ant colonies. The first studies in this field were conducted by Deneubourg et al. [2], who proposed a basic model that allowed ants to randomly move, pick up, and deposit objects in clusters according to the number of similar surrounding objects. This basic model has been successfully applied in robotics. Lumer and Faieta [3] modified the basic model into the LF algorithm, which was extended to numerical data analysis. The algorithm's basic principles are straightforward: ants are modeled as simple agents that randomly move in their environment, a square grid with periodic boundary conditions. Data items that are scattered within this environment can be picked up, transported, and dropped by the agents. The picking and dropping operations are biased by the similarity and density of the data items within the ants' local neighborhood: ants are likely to pick up data items that are either isolated or surrounded by dissimilar ones, and they tend to drop them in the vicinity of similar ones. In this way, clustering and sorting of the elements are obtained on the grid.

As a recently developed bionics optimization algorithm, the ant colony clustering algorithm possesses several advantages over traditional methods such as flexibility, robustness, decentralization, and self-organization [4–6]. These properties are well suited in distributed real-world environments. It has thus been applied in many fields such as data mining [4], graph partitioning [7], and text mining [8].

There has been a significant amount of research recently conducted on the improved performance and wider applications of ant colony clustering algorithms.

Ramos and Merelo [8] studied ant-based clustering with different ant speeds in the clustering of text documents. Wu and Shi [13] studied similarity coefficients and proposed a simpler probability conversion function. Moreover, the clustering algorithm was combined with a* K*-means method to solve document clustering. The new algorithm was called CSIM [14]. Xu et al. [15] suggested an artificial ant sleeping model (ASM) and an adaptive artificial ant clustering algorithm (A^4^C) to solve the clustering problem in data mining. In the ASM model, each datum was represented by an agent within a two-dimensional grid environment. In A^4^C, the agents formed into high-quality clusters by making simple moves based on local information within neighborhoods. An improved ant clustering algorithm called Adaptive Time-Dependent Transporter Ants (ATTA) was proposed [16] that incorporated adaptive and heterogeneous ants and time-dependent transporting activities. Yang et al. [17, 18] proposed a multi-ant colony approach for clustering data that consisted of parallel and independent ant colonies and a queen ant agent. Each ant colony had a different moving speed and probability conversion function. A hypergraph model was used to combine the results of all parallel ant colonies. Kuo et al. [19] proposed a novel clustering method called an ant* K*-means (A*K*) algorithm. The A*K* algorithm modified the* K*-means to locate objects in a cluster with probability that was updated by the pheromone, whereas the rule of the updating pheromone was based on total cluster variance. An improved ant colony optimization-based clustering technique was proposed using nearest-neighborhood interpolation, and an efficient arrhythmia clustering and detection algorithm based on a medical experiment and new ant colony clustering technique for a QRS complex was also presented [20]. Ramos et al. [21] proposed a new clustering algorithm called Hyperbox Clustering with Ant Colony Optimization (HACO) that clustered unlabeled data by placing hyperboxes in the feature spaces optimized by the ant colony optimization. A novel ant-based clustering algorithm called ACK was proposed [9] that incorporated the merits of kernel-based clustering into ant-based clustering. Tan et al. [22] proposed a simplified ant-based clustering (SABC) method based on existing research of a state-of-the-art ant-based clustering system. Tao et al. [12] redefined the distance between two data objects and improved the strategy for ants letting go and picking up data objects, thus proposing an improved ant colony clustering algorithm. Wang et al. [10] proposed an improvement to the ATTA called Logic-Based Cold Ants (LCA). In LCA, ant populations initially pick up data objects and calculate the current locations suitable for dropping; they then take the data objects not suitable for putting down directly to various objects that maximize the similarity value of the position. Moreover, to allow for the rapid formation of class cluster centers, a logic-based similarity measure was proposed in which an ant classifies objects as similar or dissimilar and groups similar objects while detaching dissimilar ones. Xu et al. [23] proposed a constrained ant clustering algorithm that was embedded with a heuristic walk mechanism based on a random walk to address constrained clustering problems that give pairs must-link and cannot-link constraints. More recently, Inkaya et al. [24] presented a novel clustering methodology based on ant colony optimization (ACO-C). In this ACO-C, two new objective functions were used that adjusted for compactness and relative separation. Each objective function evaluated the clustering solution with respect to the local characteristics of the neighborhoods.

Although many of these recently created methods appear promising, there are still shortcomings with ant colony clustering algorithms. Because ants move randomly and spend significant time finding proper places to drop or pick up objects, the computational efficiency and accuracy of ant colony clustering algorithms are low, particularly for large and complicated engineering problems. To overcome these shortcomings, a new abstraction ant colony clustering algorithm is proposed that uses a data combination mechanism. In this new algorithm, the random projections of the patterns are modified to improve computational efficiency and accuracy. The performance of the new algorithm is verified by actual datasets and compared with those of the ant colony clustering algorithm and other algorithms proposed in previous studies.

## 2. Ant Colony Clustering Algorithm and Abstraction Ant Colony Clustering Algorithm

### 2.1. Ant Colony Clustering Algorithm

To correctly describe the proposed algorithm, the basic principle underlying the ant colony clustering algorithm must be introduced.

First, data objects are randomly projected onto a single plane. Next, each ant chooses an object at random and picks up, moves, and drops the object according to a picking-up or dropping probability based on the similarity of the current object to objects in the local region. Finally, clusters are collected from the plane.

The ant colony clustering algorithm is described by the following pseudocode:

(1) Initialization: initialize the number of ants *N*, the entire number of iterations *M*, the local region side length *s*, the constant parameters *α* and *c*, and the maximum speed *v*_max_.
(2) Project the data objects onto a plane; that is, assign a random pair of coordinates (*x*, *y*) to each object.
(3) Each ant that is currently unloaded chooses an object at random.
(4) Each ant is given a random speed *v*;
(5) For *i* = 1, 2,…, *M*
   For *j* = 1,2,…, *N*
     The average similarity of all of the clustered objects is calculated.
     If the ant is unloaded, the picking-up probability *P*_*p*_ is computed. If *P*_*p*_ is greater than a random probability and an object is not simultaneously picked up by another ant, the ant picks up this object, marks itself as loaded, and moves this object to a new position; otherwise, the ant does not pick up this object and randomly selects another object.
     If the ant is loaded, the dropping probability *P*_*d*_ is computed. If *P*_*d*_ is greater than a random probability, the ant drops the object, marks itself as unloaded, and randomly selects a new object; otherwise, the ant continues moving the object to a new position.
   End
 End
(6) For *i* = 1, 2,…, *n* // for all objects [18]
   If an object is isolated (i.e., the number of neighbors it possesses is less than a given constant) then it is labeled as an outlier;
   otherwise, give this object a cluster labeling number and recursively label the same number to those objects that are neighbors of this object within the local region.
 End

The operations of the algorithm are described in detail in the following section.

#### 2.1.1. The Average Similarity Function

We assume that an ant is located at site *r* at time *t* and that it finds an object *o*_*i*_ at that site. The average similarity density of object *o*_*i*_ with the other objects *o*_*j*_ present in its neighborhood *f*(*o*_*i*_) is given by

(1) foi=max⁡0,1s2·∑oj∈Neighs×sr1−doi,ojα1+v−1/vmax,

where *α* defines a parameter used to adjust the similarity between objects. The parameter *v* defines the speed of the ants, and *v*_max_ is the maximum speed. *v* is distributed randomly in [1, *v*_max_]. Neigh_*s*×*s*_(*r*) denotes a square of *s* × *s* sites surrounding site *r*. *d*(*o*_*i*_, *o*_*j*_) is the distance between two objects *o*_*i*_ and *o*_*j*_ in the space of attributes. The Euclidean distance is used, which can be determined as

(2) $$\begin{array}{c} d\left(\;o_{i},o_{j}\;\right)=\sqrt{\overset{m}{\underset{k=1}{\sum }}{\left(o_{ik}-o_{jk}\right)}^{2}}, \end{array}$$

where *m* defines the number of attributes.

From (1), we note that the parameter *α* affects the number of clusters and the algorithm convergence rate. Objects with greater degrees of similarity have greater values of *α* and tend to cluster. Thus, the number of clusters decreases, and the algorithm becomes faster. On the contrary, if *α* is smaller, the objects have smaller degrees of similarity, and the larger group will split into smaller groups. Thus, the number of clusters will increase, and the algorithm will become slower.

#### 2.1.2. The Probability Conversion Function

The probability conversion function is a function of *f*(*o*_*i*_), and its purpose is to convert the average similarity *f*(*o*_*i*_) into picking-up and dropping probabilities. This approach is based on the following: the smaller the similarity of a data object is (i.e., fewer objects belong to the same cluster in its neighborhood), the higher the picking-up probability is, and the lower the dropping probability is. However, the larger the similarity is, the lower the picking-up probability is (i.e., objects are unlikely to be removed from dense clusters), and the higher the dropping probability is. According to this principle, the sigmoid function is used as the probability conversion function.

The picking-up probability for a randomly moving ant that is currently not carrying an object to pick up an object is given by

(3) $$\begin{array}{c} P_{p}=1-\text{Sigmoid}\left(\;f\left(\;o_{i}\;\right)\;\right), \end{array}$$

where *f*(*o*_*i*_) is the average similarity function.

Using the same method, the dropping probability for a randomly moving, loaded ant to deposit an object is given by

(4) $$\begin{array}{c} P_{d}=\text{Sigmoid}\left(\;f\left(\;o_{i}\;\right)\;\right). \end{array}$$

The sigmoid function has a natural exponential form of

(5) $$\begin{array}{c} Sigmoid\left(\;x\;\right)=\frac{1-e^{-cx}}{1+e^{-cx}}, \end{array}$$

where *c* is a slope constant that can speed up the algorithm convergence if increased.

It must be pointed out that, during the clustering procedure, some objects may exist (called outliers) with high dissimilarity to all other data elements. The outliers prevent ants from dropping them, which slows down the algorithm convergence. Here, we choose a larger parameter *c* to force the ant to drop the outliers at the later stage of the algorithm.

### 2.2. Abstraction Ant Colony Clustering Algorithm

The process behind the abstraction ant colony clustering algorithm is described as follows.

*(1) Initialization*. *n* data objects are put into *K* data reactors randomly (*K* ≤ *n*), where one data reactor is corresponding to one data type.

*(2) Iteration*. Initially, *N* ants are assigned to one data reactor, and this data reactor is the first visited data reactor. Each ant will traverse *M* (the maximum iteration step) steps to visit each data reactor. In this process, the most dissimilar data objects in each visited data reactor will be selected to be put into a suitable data reactor.

During the iteration process, each ant should abide by the following rules:

(1) If one ant visits one data reactor while only one data object exists in this data reactor, this data object will be picked up with probability 1 to be dropped at suitable data reactor.

(2) If an ant is not loading a data object and the visited data reactor contains more than one data object, then the average similarity *f*(*o*_*i*_) of all the data objects *o*_*i*_  (*o*_*i*_ ∈ cluster) in the current data reactor (the similarity of one data object to the other data object in the current data reactor) is computed. The ant picks up the most dissimilar data object with probability *p*_*p*−mean_(*o*_*i*_) and randomly visits another data reactor.

The average similarity *f*(*o*_*i*_) of the data object *o*_*i*_ in the current data reactor can be described as

(6) foi=1s−1∑oj∈clusteroi1−doi,ojα,if  f>0,0,otherwise,

where *f*(*o*_*i*_) is the average similarity of data *o*_*i*_ to other data objects *o*_*j*_  (*o*_*j*_ ≠ *o*_*i*_, *o*_*j*_ ∈ cluster) in the current data reactor, *s* is the number of data objects in the data reactor visited by the current ant, cluster(*o*_*i*_) is the data reactor that the data object *o*_*i*_ belongs to, *d*(*o*_*i*_, *o*_*j*_) is the Euclidean distance, and *α* defines a parameter used to adjust the similarity between objects.

The picking-up probability of a data object *o*_*i*_ in the current data reactor can be described as

(7) $$\begin{array}{c} p_{p}\left(\;o_{i}\;\right)={\left(\;\frac{k_{p}}{k_{p}+f\left(o_{i}\right)}\;\right)}^{2}, \end{array}$$

where *k*_*p*_ is a threshold for picking up one data object.

If *f*(*o*_*i*_) ≪ *k*_*p*_, then *p*_pick off_ ≈ 1. This is to say that the ant will pick up this data object, which is not similar to other data objects in this data reactor, with a very high probability. On the contrary, if *f*(*o*_*i*_) ≫ *k*_*p*_, then *p*_pick off_ ≈ 0, which shows that the object *o*_*i*_ is similar to other data objects in data reactor, and this object has a very small probability of being picked up.

(3) If an ant that has loaded the data object *o*_*i*_ visits one data reactor that contains more than one data object, the ant will place the data object into the current data reactor, and the “average similarity” of all the data objects in the current data reactor can be computed. Next, the most dissimilar data objects in the current data reactor will be picked up with a probability *p*_*p*−mean_(*o*_*i*_). Finally, the ant loads this new data object and visits the next data reactor.

(4) If an ant with one data object loaded has not found the data reactor to drop the data object after *M* steps, the ant will construct a new data reactor to place the data object into.

When the number of clustering types is larger than the practical number of data object types, one principle of data reactor combination is applied. Before the most dissimilar data object in the current data reactor visited by the ant is selected, the current data reactor will be compared with the other data reactors, and the data reactors that are similar to a given degree will be combined with some probability.

The combination probability of data reactors can be described as

(8) pcombinei=2 similarci,cj,if  similarci,cj<kc;1,otherwise,

where similar(*c*_*i*_, *c*_*j*_) is the similarity function of the two data reactors *i* and *j*, which can be described as

(9) $$\begin{array}{c} similar\left(\;c_{i},c_{j}\;\right)=1-\frac{d\left(c_{i},c_{j}^{}\right)}{\alpha_{1}}, \end{array}$$

where *d*(*c*_*i*_, *c*_*j*_) is the Euclidean distance between the two data reactors' centers, *c*_*i*_ is the center of data reactor *i* and *c*_*j*_ is the center of data reactor *j*, *α*_1_ is a parameter used to adjust the similarity between data objects, and *k*_*c*_ is a threshold parameter.

If similar(*c*_*i*_, *c*_*j*_) ≪ *k*_*c*_, the combination probability will be *p*_combine_ ≈ 0, and if similar(*c*_*i*_, *c*_*j*_) ≫ *k*_*c*_, the combination probability will be 1.

*(3) Termination*. The termination condition is that the difference of the clustering results for neighboring iterations is less than 10*e* − 5.

The flowchart of the abstraction ant colony clustering algorithm is as shown in Figure 1.

Because the combination mechanism for data reactors used in the proposed new algorithm is the abstraction of clustering mechanism of similar data used in traditional ant colony clustering algorithms, the proposed new algorithm is called the abstraction ant colony clustering algorithm.

## 3. Applications

To verify the abstraction ant colony clustering algorithm and to compare it with other clustering algorithms, some classical datasets are used.

### 3.1. Iris Dataset

The Iris dataset is constructed using data that describe the features of iris plants. The dataset contains 150 instances with three classes of 50 instances each, where each class refers to a type of iris plant. To each instance, there are four attributes. The three classes are* Iris setosa*,* Iris versicolour*, and* Iris virginica*. The four attributes describe the flower of iris plants, which are the sepal length, sepal width, petal length, and petal width. The Iris dataset was created by Fisher in July 1988 and is perhaps the best known dataset in the pattern recognition literature. A detailed description of this dataset can be found at http://archive.ics.uci.edu/ml/datasets/Iris.

For comparison purposes, the traditional* K*-means algorithm [25], the ant colony clustering algorithm, and the abstraction ant colony clustering algorithm are all applied to this dataset. In this example, the dataset consists of numeric-type data; therefore, the* K*-means algorithm is used.

In this study, the clustering accuracy is computed by the following equation:

(10) r=1−number  of  samples  that  is  classified  mistakenlynumber  of  all  samples.

For the dataset whose clustering results are known previously, such as the datasets used in this study, the number of samples that is classified mistakenly can be obtained easily through comparisons of the clustering results by the clustering method with the known clustering results. This idea is used by many researchers in their studies [9–12]. For example, Zhang and Cao [9] defined one evaluation function to evaluate the performance of the clustering algorithms, which is called “error rate (ER),” using this idea. Moreover, Hatamlou [11] proposed one criterion to evaluate the performance of the clustering algorithms, which is also called “error rate (ER),” using this idea. But the definitions of two ERs are different.

It must be pointed out that, in the tables of the results, the “number of samples that is classified mistakenly” is simplified to “mistaken partition numbers.”

Based on testing and experience, the parameters of the ant colony clustering algorithm and the abstraction ant colony clustering algorithm are as follows.

For the ant colony clustering algorithm,

(11) N=20,M=15000,s=3,α=1.5,vmax=0.85,c=3.

For the abstraction ant colony clustering algorithm,

(12) M=15000,kp=0.1,kc=0.15,α=1.5,N=20,α1=0.4,s=3.

Using these parameters, the clustering results of 20 random tests using the three algorithms are shown in Table 1.

As seen in Table 1, the average number of iteration steps of the ant colony clustering algorithm is lower than that of the* K*-means algorithm, and the average number of iteration steps of the abstraction ant colony clustering algorithm is lower than that of the ant colony clustering algorithm. Therefore, the abstraction ant colony clustering algorithm has the fastest average iteration speed. The average processing time of the abstraction ant colony clustering algorithm (32.52 s) is faster than the ant colony clustering algorithm (36.86 s) but slower than the* K*-means algorithm (26.61 s). Therefore, although the computational efficiency of the abstraction ant colony clustering algorithm is better than that of the ant colony clustering algorithm, the* K*-means algorithm is more efficient. However, the* K*-means algorithm requires* a priori* knowledge of the number of clusters. In this study, it is provided with the correct number of clusters. Thus, it is unfair to compare the processing times of the two ant colony clustering algorithms with that of the* K*-means algorithm. Moreover, the average accuracy of the ant colony clustering algorithm is 90.43%, compared to 81.61% for the* K*-means algorithm and 96.34% for the abstraction ant colony clustering algorithm. The computational accuracy of the abstraction ant colony clustering algorithm is superior to the ant colony clustering algorithm and the* K*-means clustering algorithm. Based on the gap between the minimum and maximum mistaken partition numbers, the computing stability of the abstraction ant colony clustering algorithm is superior to the others, with 8 mistaken partition numbers for the abstraction ant colony clustering algorithm, 16 for the ant colony clustering algorithm, and 26 for the* K*-means algorithm.

To compare the computational effects of the ant colony clustering algorithm and the abstraction ant colony clustering algorithm with other clustering algorithms used in previous studies, the average accuracy for each algorithm is summarized in Table 2.

As seen in Table 2, the average accuracy of the abstraction ant colony clustering algorithm, at 96.34%, is the best for all fourteen algorithms. The second-best result is 95.03% for the ACK algorithm [9], whereas the worst result is 89.94% for the particle swarm optimization method [11]. Moreover, the average accuracies of all ant colony-based clustering algorithms are greater than 90%, whereas the results of other algorithms are all less than 90%. Therefore, algorithms based on ant colonies outperform other algorithms such as particle swarm optimization, Big Bang-Big Crunch, gravitational search, and black hole algorithms. In examining the results of all ant colony-based clustering algorithms, the best average accuracy was 96.34% for the abstraction ant colony clustering algorithm proposed in this study. The worst ant colony-based average accuracy was 90% for the LAC algorithm [10].

In assessing the last three algorithms in Table 2, the average accuracy of the abstraction ant colony clustering algorithm is the best, but the difference between the highest accuracy and lowest accuracy, at 5.33%, is not the least; this distinction belongs to the improved ant colony clustering algorithm [12], at 1%. This means that the computational stability of the improved ant colony clustering algorithm is the best. Because the highest accuracy and lowest accuracy values for the other referenced algorithms were not available, their computational stabilities cannot be analyzed.

### 3.2. Animal Dataset

The Animal, or Zoo database, dataset was created by Forsyth in May 1990. The dataset contains 101 instances and 7 classes as well as a simple database containing 17 Boolean-valued attributes. The “type” attribute appears to be the class attribute. A detailed description of this dataset can be found at http://archive.ics.uci.edu/ml/datasets/Zoo.

For comparison purposes, the traditional* K-*modes algorithm [26], ant colony clustering algorithm, and abstraction ant colony clustering algorithm are all applied to this dataset. In this example, the dataset consists of the Boolean type data; therefore, the* K*-modes algorithm is used.

In this study, the clustering accuracy is also computed by (10).

Based on testing and experience, the parameters of the ant colony clustering algorithm and the abstraction ant colony clustering algorithm are as follows.

For the ant colony clustering algorithm,

(13) N=30,M=15000,s=3,α=0.7,vmax=0.85,c=5.

For the abstraction ant colony clustering algorithm,

(14) M=15000,kp=0.2,kc=0.05,α=0.7,N=20,α1=0.5,s=3.

Based on these parameters, the clustering results of 20 random tests using the three algorithms are given in Table 3.

As seen in Table 3, the average iteration steps of the abstraction ant colony clustering algorithm are the least, which is 8673.73, the second is that of the ant colony clustering algorithm, and the biggest is that using* K*-modes algorithm, which is 31524.56. The average processing time of the* K*-modes algorithm (27.71 s) is the least, the second is that of the abstraction ant colony clustering algorithm (34.36 s), and the biggest is that using ant colony clustering algorithm (37.52 s). Therefore, although the computational efficiency of the abstraction ant colony clustering algorithm is better than that of the ant colony clustering algorithm, the* K*-modes algorithm is more efficient. However, the* K*-modes algorithm requires* a priori* knowledge of the number of clusters. In this study, it is provided with the correct number of clusters. Thus, it is unfair to compare the processing times of the two ant colony clustering algorithms with that of the* K*-modes algorithm. Moreover, the average accuracy of the ant colony clustering algorithm is 89.7%, compared to 83.17% for the* K*-modes algorithm and 93.74% for the abstraction ant colony clustering algorithm. Therefore, the computational accuracy of the abstraction ant colony clustering algorithm is superior to the ant colony clustering algorithm and the* K*-modes clustering algorithm. Based on the gap between the minimum and maximum mistaken partition numbers, the computing stability of the abstraction ant colony clustering algorithm is the best, with 15 mistaken partition numbers, while that of the* K*-modes clustering algorithm is the poorest, with 26 mistaken partition numbers.

To compare the computational effects of the ant colony clustering algorithm and the abstraction ant colony clustering algorithm with other clustering algorithms used in previous studies, the average accuracy for each algorithm is summarized in Table 4.

As seen in Table 4, in the eight algorithms, all algorithms are from ant colony algorithm. And the average accuracy of the abstraction ant colony clustering algorithm, at the 93.74%, is the best. The second-best is 89.7% for the ant colony clustering algorithm, whereas the worst result is 78.24% for the LF algorithm. Therefore, in those ant colony-based clustering algorithms, the computational results of the abstraction ant colony clustering algorithm are the best.

### 3.3. Soybean (Small) Dataset

The Soybean dataset is Michalski's famous Soybean disease database, which was donated in 1987. This dataset contains 47 instances, and each instance is described using 35 attributes. All attributes appear with numeric values. The dataset contains four classes with instances of 10, 10, 10, and 17.

A detailed description of this dataset can be found at http://archive.ics.uci.edu/ml/datasets/Soybean+(Small).

For comparison purposes, the traditional* K-*means algorithm, the ant colony clustering algorithm, and the abstraction ant colony clustering algorithm are all applied to this dataset. In this example, the dataset also consists of numeric-type data; therefore, the* K*-means algorithm is used too.

In this study, the clustering accuracy is also computed by (10).

Based on testing and experience, the parameters of the ant colony clustering algorithm and the abstraction ant colony clustering algorithm are as follows.

For the ant colony clustering algorithm,

(15) N=50,M=15000,s=3,α=0.6,vmax=0.85,c=6.

For the abstraction ant colony clustering algorithm,

(16) M=15000,kp=0.1,kc=0.15,α=0.5,N=20,α1=0.3,s=3.

Based on these parameters, the clustering results of 20 random tests using the three algorithms for the Soybean dataset are as shown in Table 5.

As seen in Table 5, the average iteration steps of the abstraction ant colony clustering algorithm are the least, which is 7235.25, the second is that of the ant colony clustering algorithm, which is 13785.22, and the biggest is that using* K*-means algorithm, which is 23342.43. Therefore, the iteration steps of the abstraction ant colony clustering algorithm are the best. The average processing time of the* K*-means algorithm (34.39 s) is the least, the second is that of the abstraction ant colony clustering algorithm (47.42 s), and the biggest one is that using the ant colony clustering algorithm (52.35 s). Therefore, although the computational efficiency of the abstraction ant colony clustering algorithm is better than that of the ant colony clustering algorithm, the* K*-means algorithm is more efficient. However, the* K*-means algorithm requires* a priori* knowledge of the number of clusters. In this study, it is provided with the correct number of clusters. Thus, it is unfair to compare the processing times of the two ant colony clustering algorithms with that of the* K*-means algorithm. Moreover, the average accuracy of the ant colony clustering algorithm is 92.51% compared to 84% for the* K*-means algorithm and 97.35% for the abstraction ant colony clustering algorithm. Therefore, the computational accuracy of the abstraction ant colony clustering algorithm is superior to the ant colony clustering algorithm and the* K*-means clustering algorithm. Based on the gap between the minimum and maximum mistaken partition numbers, the one for the abstraction ant colony clustering algorithm is the least, which is 14, while the one for the* K*-means clustering algorithm is the biggest, which is 23. Therefore, the computing stability of the abstraction ant colony clustering algorithm is the best. It is clear that the abstraction ant colony clustering algorithm can solve this problem with a high degree of accuracy and speed.

To compare the computational effects of the ant colony clustering algorithm and the abstraction ant colony clustering algorithm with other clustering algorithms used in previous studies, the average accuracy for each algorithm is summarized in Table 6.

As seen in Table 6, the result by the LCA algorithm is the best, whose average accuracy is 100%. The second result is 97.35%, which is using the abstraction ant colony clustering algorithm. The worst result is 92.51%, which is by the ant colony clustering algorithm. It is clear that, for this dataset, in three algorithms from the ant colony algorithm, the computational results of the abstraction ant colony clustering algorithm are not the best.

Therefore, the abstraction ant colony clustering algorithm can solve the clustering problem with a high degree of accuracy and speed. However, its results are the best for the most problems but not for all problems.

### 3.4. Yeast Dataset

The Yeast dataset contains 1484 instances and each instance is described using 8 attributes. All attributes appear with numeric values. The dataset contains 10 classes with instances of 463, 429, 244, 163, 51, 44, 35, 30, 200, and 5. A detailed description of this dataset can be found at http://archive.ics.uci.edu/ml/datasets/Yeast.

For comparison purposes, the traditional* K-*means algorithm, the ant colony clustering algorithm, and the abstraction ant colony clustering algorithm are all applied to this dataset.

In this study, the clustering accuracy is also computed using (10).

Based on testing and experience, the parameters of the ant colony clustering algorithm and the abstraction ant colony clustering algorithm are as follows.

For the ant colony clustering algorithm,

(17) N=55,M=15000,s=3,α=0.45,vmax=0.85,c=8.

For the abstraction ant colony clustering algorithm,

(18) M=15000,kp=0.05,kc=0.25,α=0.45,N=20,α1=0.25,s=3.

Based on these parameters, the clustering results of 20 random tests using the three algorithms are given in Table 7.

As seen in Table 7, the average processing time of the* K*-means algorithm (54.34 s) is the least, the second is that of the abstraction ant colony clustering algorithm (105.31 s), and the biggest is that using the ant colony clustering algorithm (123.57 s). Therefore, the computational efficiency of the abstraction ant colony clustering algorithm is better than that of the ant colony clustering algorithm. Moreover, the average accuracy of ant colony clustering algorithm is 82.86% compared to 75.42% for the* K*-means algorithm and 88.56% for the abstraction ant colony clustering algorithm. Therefore, the computational accuracy of the abstraction ant colony clustering algorithm is superior to the ant colony clustering algorithm and the* K*-means clustering algorithm. Based on the gap between the minimum and maximum mistaken partition numbers, the one for the abstraction ant colony clustering algorithm is the least, which is 166, while the one for the* K*-means clustering algorithm is the biggest, which is 354. Therefore, the computing stability of the abstraction ant colony clustering algorithm is the best.

## 4. Sensitivity Analysis of Main Parameters for Abstraction Ant Colony Clustering Algorithm and Ant Colony Clustering Algorithm

To conduct a sensitivity analysis of the main parameters for the abstraction ant colony clustering algorithm and the ant colony clustering algorithm, the Iris dataset is applied in this study.

### 4.1. Abstraction Ant Colony Clustering Algorithm

The parameters *k*_*p*_, *k*_*c*_, *α*_1_, and *α* are analyzed because they significantly influence the abstraction ant colony clustering algorithm. In this study, the convergence speed is represented by the iterations number and the computation performance is represented by the clustering accuracy.

The relationship between *k*_*p*_ and the convergence speed of the algorithm is shown in Figure 2. The relationship between *k*_*p*_ and the performance of the algorithm is shown in Figure 3.

Based on Figures 2 and 3, the relationship between *k*_*p*_ and convergence speed is a monotonic function, whereas its relationship with computation performance is a unimodal function. As *k*_*p*_ increases, the computation speed and amplitude will increase. However, the variable law of computation performance is complex. If *k*_*p*_ is less than 0.1, then computation performance will improve as *k*_*p*_ increases, whereas if *k*_*p*_ is greater than 0.1, then computation performance will decline as *k*_*p*_ increases.

The relationship between *k*_*c*_ and the convergence speed of the algorithm is shown in Figure 4. The relationship between *k*_*c*_ and the performance of the algorithm is shown in Figure 5.

As seen in Figure 4, the relationship between *k*_*c*_ and the convergence speed is approximately defined as a downward straight line; that is, as *k*_*c*_ increases, the computation speed will decrease. As seen in Figure 5, the relationship between *k*_*c*_ and computation performance is a unimodal function. When *k*_*c*_ is less than 0.15, computation performance will decline as *k*_*c*_ increases, whereas when *k*_*c*_ is greater than 0.15, computation performance will improve as *k*_*c*_ increases.

The relationship between *α*_1_ and the convergence speed of the algorithm is shown in Figure 6. The relationship between *α*_1_ and the performance of the algorithm is shown in Figure 7.

As seen in Figure 6, the relationship between *α*_1_ and the convergence speed is defined as an upward straight line; that is, as *α*_1_ increases, the computation speed increases. As seen in Figure 7, the relationship between *α*_1_ and computation performance is a unimodal function. When *α*_1_ is less than 0.4, computation performance improves as *α*_1_ increases, whereas when *α*_1_ is greater than 0.4, computation performance declines as *α*_1_ increases.

The relationship between *α* and the convergence speed of the algorithm is shown in Figure 8. The relationship between *α* and the performance of the algorithm is shown in Figure 9.

As seen in Figures 8 and 9, the relationship between *α* and the convergence speed is a monotonic function; that is, as *α* increases, the iterations number will decrease or the computation speed will increase. However, the relationship between *α* and computation performance is a unimodal function. When *α* is less than 1.5, the clustering accuracy increases as *α* increases; that is, the computation performance will improve. When *α* is greater than 1.5, the clustering accuracy will decrease as *α* increases; that is, the computation performance will decline.

### 4.2. Ant Colony Clustering Algorithm

The parameters *N*, *α*, and *c* are analyzed because they significantly influence the ant colony clustering algorithm.

The relationship between *N* and the convergence speed of the algorithm is shown in Figure 10. The relationship between *N* and the performance of the algorithm is shown in Figure 11.

Based on Figures 10 and 11, the relationship between *N* and convergence speed is approximately a straight line, whereas its relationship with computation performance is a unimodal function. As *N* increases, the computation speed will increase. However, the variable law of computation performance is complex. As seen in Figure 7, if *N* is less than 20, then computation performance will improve as *N* increases, whereas if *N* is greater than 20, then computation performance will decline as *k*_*p*_ increases. Therefore, for this example, the suitable value of *N* should be 20.

The relationship between *α* and the convergence speed of the algorithm is shown in Figure 12. The relationship between *α* and the performance of the algorithm is shown in Figure 13.

As seen in Figures 12 and 13, the relationship between *α* and the convergence speed is one monotonic function; that is, as *α* increases, the iterations number will decrease or the computation speed will increase. However, the relationship between *α* and the computation performance is unimodal function. When *α* is less than 1.5, the clustering accuracy will increase as *α* increases; that is, the computation performance will be better. When *α* is bigger than 1.5, the clustering accuracy will decrease as *α* increases; that is, the computation performance will be poorer. Therefore, for this example, the suitable value of *α* is 1.5.

The relationship between *c* and the convergence speed of the algorithm is shown in Figure 14. The relationship between *c* and the performance of the algorithm is shown in Figure 15.

As seen in Figures 14 and 15, the relationship between *c* and the convergence speed is one monotonic function. As *c* increases, the computation speed will increase too. But at the same time, the increasing amplitude of computation speed will decrease. The turning point is near *c* = 2.5. However, the relationship between *c* and the computation performance is also a unimodal function. When *c* is less than 3.5, the clustering accuracy will increase as *c* increases; that is to say, the computation performance will be better. When *c* is bigger than 3.5, the clustering accuracy will decrease as *c* increases; that is to say, the computation performance will be poorer. Therefore, for this example, the suitable value of *c* is 3.

Comparing the analysis results of two algorithms, the following conclusions can be drawn. The influence law of parameter *N* for the ant colony clustering algorithm is similar to those of parameters *α*_1_ and *k*_*p*_ for the abstraction ant colony clustering algorithm. Moreover, the influence laws of parameter *α* for the ant colony clustering algorithm and the abstraction ant colony clustering algorithm are similar. The influence law of parameter *α* on the convergence speed for the ant colony clustering algorithm is similar to that of parameter *k*_*c*_ for the abstraction ant colony clustering algorithm. However, the influence laws on the computation performance for two parameters are completely opposite. Moreover, the influence law of parameter *c* for the ant colony clustering algorithm is similar to those of parameters *α* for the abstraction ant colony clustering algorithm.

## 5. Conclusions

Clustering analysis is an important tool and descriptive task used in many disciplines and applications to identify homogeneous groups of objects based on the values of their attributes. The ant colony clustering algorithm is a swarm-intelligent method for solving clustering problems that is inspired by the behavior of ant colonies in clustering their corpses and sorting their larvae. This algorithm can solve complicated clustering problems very well. However, the ant colony clustering algorithm exhibits several shortcomings with large complicated engineering problems such as poor computational efficiency and accuracy. To overcome these shortcomings, a new abstraction ant colony clustering algorithm using a data combination mechanism is proposed. Using three actual datasets (an Iris dataset, a Zoo dataset, and a Soybean dataset), the performance of the new algorithm is verified and compared with an ant colony clustering algorithm and other algorithms proposed in previous studies. The results show that the abstraction ant colony clustering algorithm can solve the clustering problem with a high degree of accuracy and speed while providing very good computing stability. For most datasets, the abstraction ant colony clustering algorithm results are superior. In other words, the computational accuracy of the abstraction ant colony clustering algorithm is superior to the ant colony clustering algorithm and other algorithms proposed in previous studies. In addition, the sensitivity of the main parameters for the abstraction ant colony clustering algorithm and the ant colony clustering algorithm is analyzed using Iris dataset to gauge their influence on convergence speed and computation performance.

However, the numbers of parameters for the two ant colony clustering algorithms are large and must be selected through testing and experience. This is the major limitation of ant colony clustering algorithms.

Based on the analysis results in this study, the following suggestions can be offered to conveniently select parameters. As for the main parameters that significantly affect the algorithms, such as *k*_*p*_, *k*_*c*_, *α*_1_, and *α* for the abstraction ant colony clustering algorithm or *N*, *α*, and *c* for the ant colony clustering algorithm, these can be determined by trials based on sensitivity analysis results for the specific datasets. Because the influence laws of parameters for different datasets are similar, according to the influence laws from the sensitivity analysis results shown in this study, the suitable values of parameters can be determined by trials. The selection process for the suitable values may be as follows. Firstly, the initial values of parameters should be guessed from the previous experience or the studies. Thus, the values can be changed by trials according to the influence laws. Finally, the suitable values can be obtained through some trials. As a result, the values of the main parameters should be different for different datasets. Conversely, other parameters that barely affect the algorithms can be determined through testing and experience, and these parameters can be fixed for different datasets. For example, the value of *s* can be fixed as 3 or 4 for different datasets, similar to *s* = 3 being used in this study for the three different datasets.

## Figures and Tables

**Figure 1:**
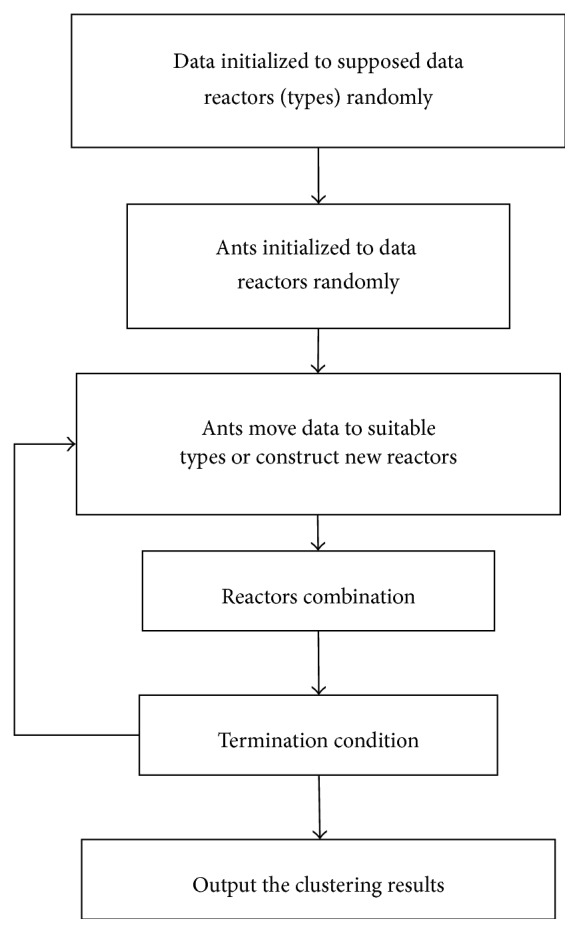
Flowchart of the abstraction ant colony clustering algorithm.

**Figure 2:**
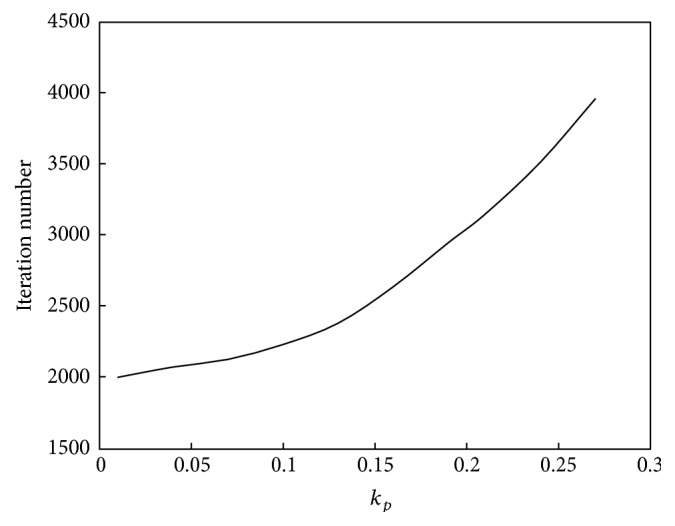
Relationship between *k*_*p*_ and convergence speed of the abstraction ant colony clustering algorithm.

**Figure 3:**
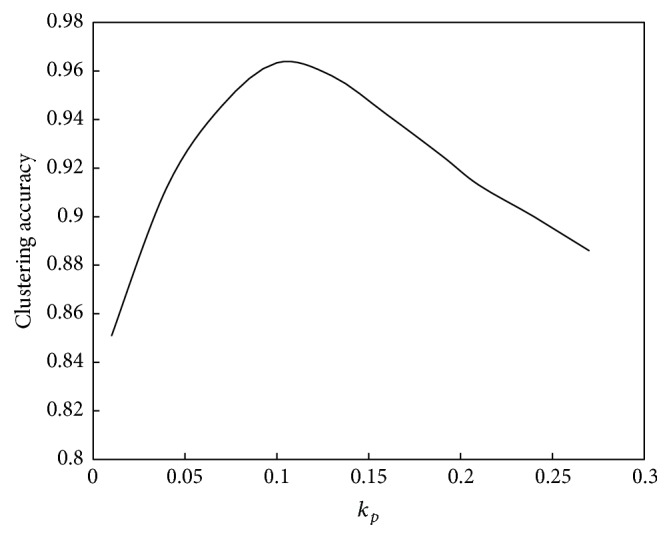
Relationship between *k*_*p*_ and performance of the abstraction ant colony clustering algorithm.

**Figure 4:**
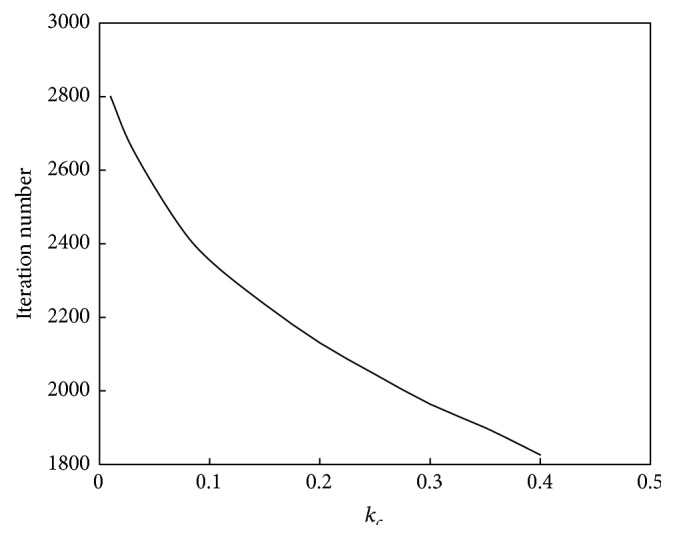
Relationship between *k*_*c*_ and convergence speed of the abstraction ant colony clustering algorithm.

**Figure 5:**
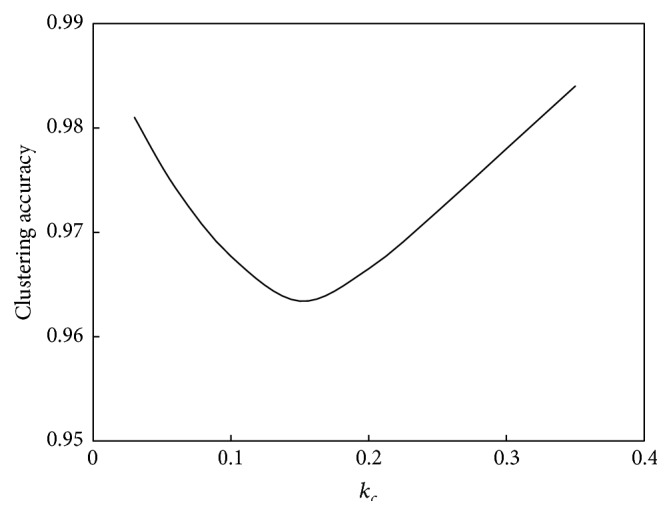
Relationship between *k*_*c*_ and performance of the abstraction ant colony clustering algorithm.

**Figure 6:**
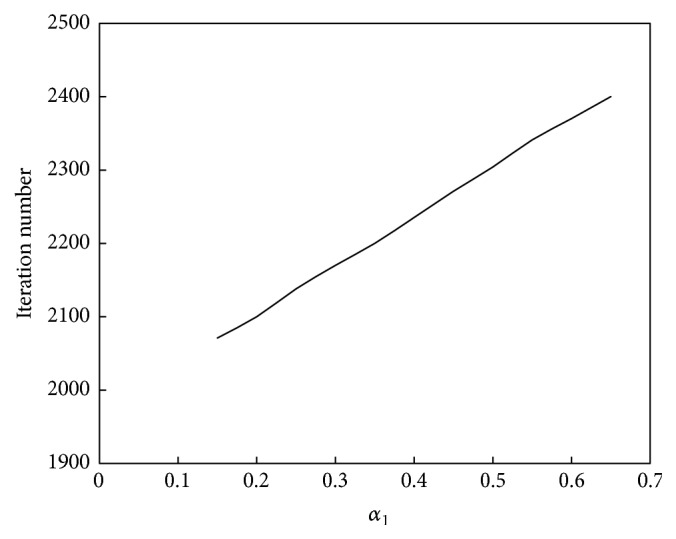
Relationship between *α*_1_ and convergence speed of the abstraction ant colony clustering algorithm.

**Figure 7:**
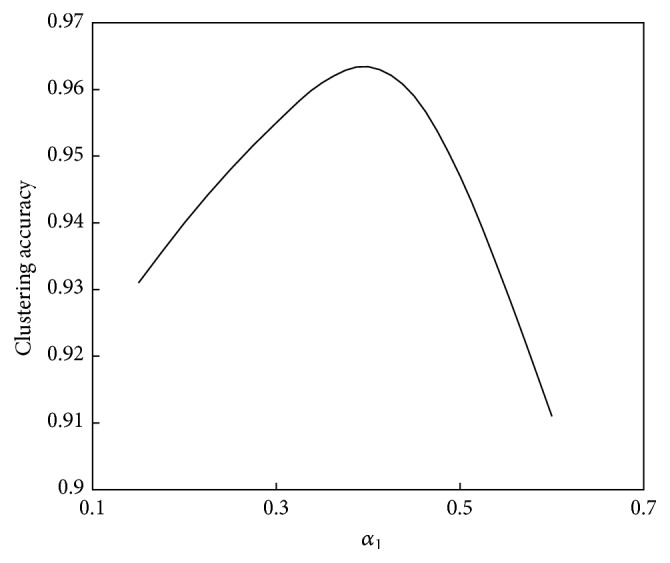
Relationship between *α*_1_ and performance of the abstraction ant colony clustering algorithm.

**Figure 8:**
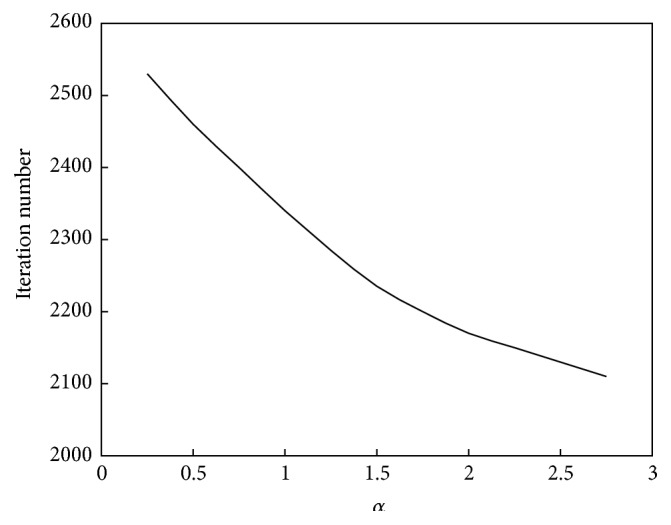
Relationship between *α* and convergence speed of the abstraction ant colony clustering algorithm.

**Figure 9:**
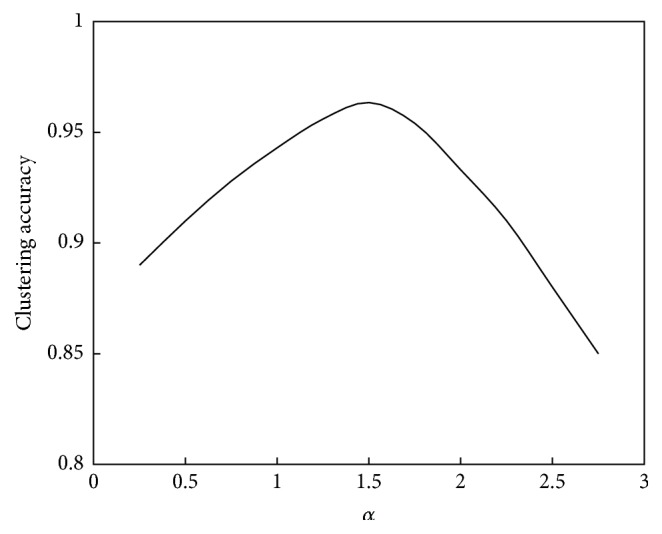
Relationship between *α* and performance of the abstraction ant colony clustering algorithm.

**Figure 10:**
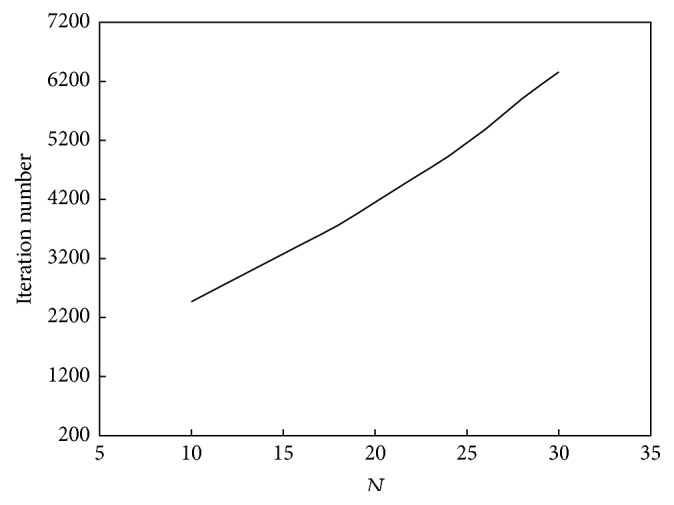
Relationship between *N* and convergence speed of the ant colony clustering algorithm.

**Figure 11:**
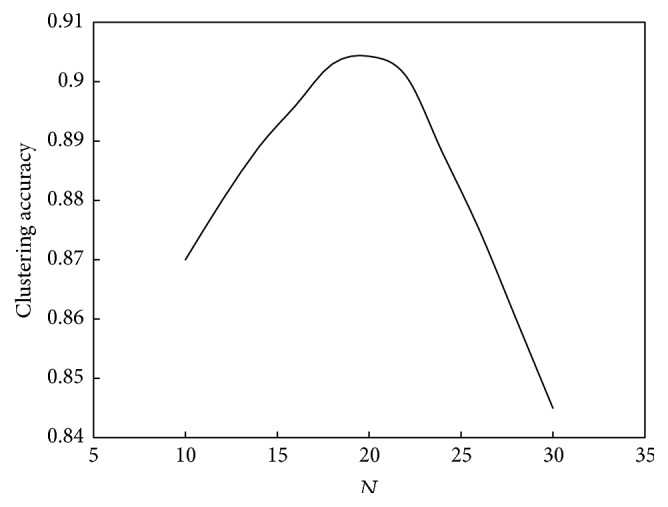
Relationship between *N* and performance of the ant colony clustering algorithm.

**Figure 12:**
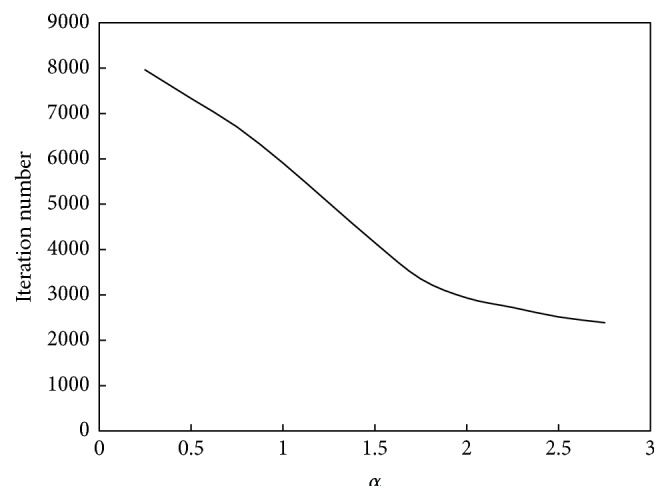
Relationship between *α* and convergence speed of the ant colony clustering algorithm.

**Figure 13:**
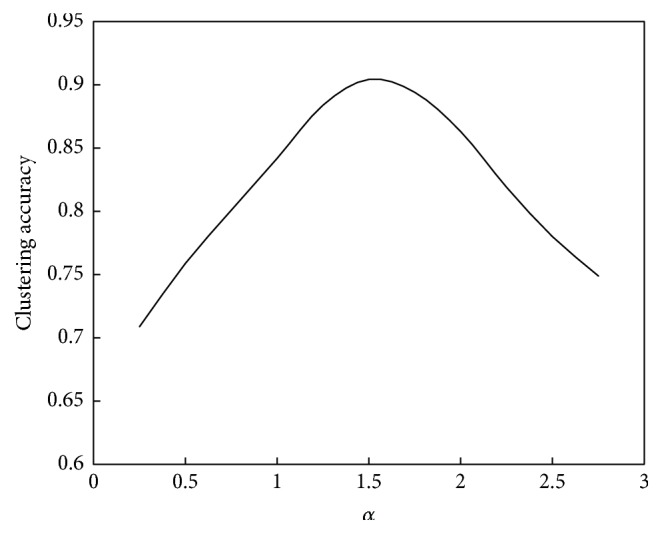
Relationship between *α* and performance of the ant colony clustering algorithm.

**Figure 14:**
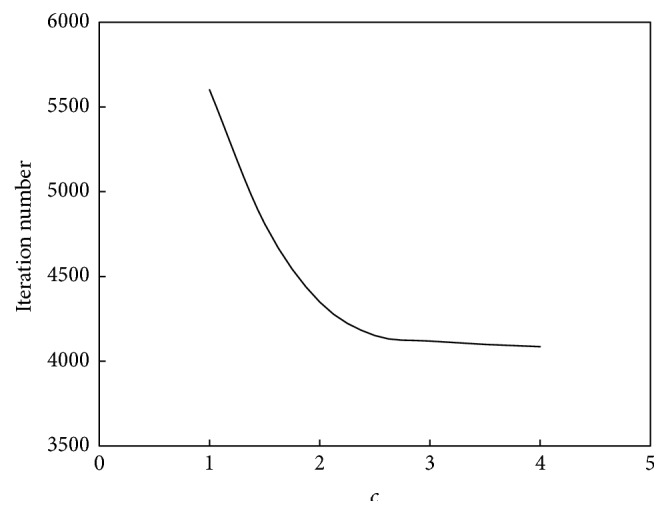
Relationship between *c* and convergence speed of ant colony clustering algorithm.

**Figure 15:**
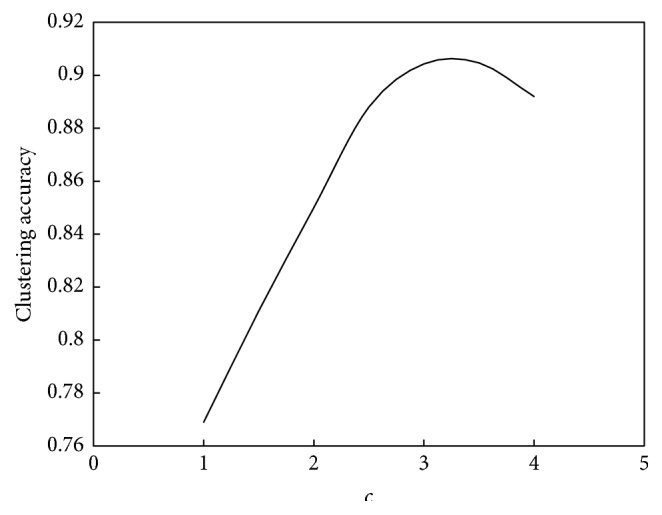
Relationship between *c* and performance of ant colony clustering algorithm.

**Table 1:** Clustering results of 20 random tests for Iris dataset.

	*K*-means algorithm	Ant colony clustering algorithm	Abstraction ant colony clustering algorithm
Average iteration steps of running 20 cycles	6342.43	4151.4	2235.25
Average processing time of running 20 cycles (s)	26.61	36.86	32.53
Minimum mistaken partition numbers	15	9	3
Maximum mistaken partition numbers	41	25	11
Average mistaken partition numbers	27.59	14.36	5.49
Average accuracy of running 20 cycles (%)	81.61	90.43	96.34

**Table 2:** Comparison of computational effect for Iris dataset.

Clustering algorithms	Average accuracy (%)	Lowest accuracy (%)	Highest accuracy (%)
LF algorithm [9]	90.34	—	—
ATTA [9]	91.57	—	—
ACP [9]	91.41	—	—
ACP-F [9]	94.17	—	—
ACK-I [9]	93.04	—	—
ACK [9]	95.03	—	—
LCA [10]	90	—	—
Particle swarm optimization [11]	89.94	—	—
Big Bang-Big Crunch algorithm [11]	89.95	—	—
Gravitational search algorithm [11]	89.96	—	—
Black hole algorithm [11]	89.98	—	—
Improved ant colony clustering algorithm [12]	91	92	93
Ant colony clustering algorithm in this study	90.43	83.33	94
Abstraction ant colony clustering algorithm in this study	96.34	92.67	98

**Table 3:** Clustering results of 20 random tests for Animal dataset.

	*K*-modes algorithm	Ant colony clustering algorithm	Abstraction ant colony clustering algorithm
Average iteration steps of running 20 cycles	31524.56	12425.52	8673.73
Average processing time of running 20 cycles (s)	27.71	37.52	34.36
Minimum mistaken partition numbers (including Mammalia)	6	4	4
Maximum mistaken partition numbers (including Mammalia)	32	25	19
Average mistaken partition numbers (including Mammalia)	17	10.4	6.32
Average accuracy of running 20 cycles (%)	83.17	89.7	93.74

**Table 4:** Comparison of computational effect for Animal dataset.

Clustering algorithms	Average accuracy (%)
LF algorithm [9]	78.24
ATTA [9]	88.84
ACP [9]	79.74
ACP-F [9]	87.23
ACK-I [9]	81.8
ACK [9]	87.3
Ant colony clustering algorithm in this study	89.7
Abstraction ant colony clustering algorithm in this study	93.74

**Table 5:** Clustering results of 20 random tests for Soybean (Small) dataset.

	*K*-means algorithm	Ant colony clustering algorithm	Abstraction ant colony clustering algorithm
Average iteration steps of running 20 cycles	23342.43	13785.22	7235.25
Average processing time of running 20 cycles (s)	34.39	52.35	47.42
Minimum mistaken partition numbers	5	4	3
Maximum mistaken partition numbers	28	21	17
Average mistaken partition numbers	15.65	10.37	7.37
Average accuracy of running 20 cycles (%)	84	92.51	97.35

**Table 6:** Comparison of computational effect for Soybean dataset.

Clustering algorithms	Average accuracy (%)
LCA [10]	100
Ant colony clustering algorithm in this study	92.51
Abstraction ant colony clustering algorithm in this study	97.35

**Table 7:** Clustering results of 20 random tests for Yeast dataset.

	*K*-means algorithm	Ant colony clustering algorithm	Abstraction ant colony clustering algorithm
Average processing time of running 20 cycles (s)	54.34	123.57	105.31
Minimum mistaken partition numbers	178	102	73
Maximum mistaken partition numbers	532	412	239
Average mistaken partition numbers	364.77	254.36	169.77
Average accuracy of running 20 cycles (%)	75.42	82.86	88.56
